# Genetic characterization of multidrug-resistant *Escherichia coli* harboring colistin-resistant gene isolated from food animals in food supply chain

**DOI:** 10.3389/fcimb.2024.1289134

**Published:** 2024-02-02

**Authors:** Peechanika Chopjitt, Parichart Boueroy, Masatomo Morita, Tetsuya Iida, Yukihiro Akeda, Sihigeyuki Hamada, Anusak Kerdsin

**Affiliations:** ^1^ Faculty of Public Health, Kasetsart University, Sakon Nakhon, Thailand; ^2^ Department of Bacteriology I, National Institute of Infectious Diseases, Tokyo, Japan; ^3^ Japan-Thailand Research Collaboration Center on Emerging and Re-emerging Infections, Research Institute for Microbial Diseases, Osaka University, Osaka, Japan; ^4^ Department of Bacterial Infections, Research Institute for Microbial Diseases, Osaka University, Osaka, Japan; ^5^ Center for Infectious Disease Education and Research, Osaka University, Osaka, Japan

**Keywords:** colistin-resistant *E. col*, *mcr* gene, plasmid replicon typing, foods, IncX4, IncP1, IncI2

## Abstract

Colistin is widely used for the prophylaxis and treatment of infectious disease in humans and livestock. However, the global food chain may actively promote the dissemination of colistin-resistant bacteria in the world. Mobile colistin-resistant (*mcr*) genes have spread globally, in both communities and hospitals. This study sought to genomically characterize *mcr*-mediated colistin resistance in 16 *Escherichia coli* strains isolated from retail meat samples using whole genome sequencing with short-read and long-read platforms. To assess colistin resistance and the transferability of *mcr* genes, antimicrobial susceptibility testing and conjugation experiments were conducted. Among the 16 isolates, 11 contained *mcr-1*, whereas three carried *mcr-3* and two contained *mcr-1* and *mcr-3*. All isolates had minimum inhibitory concentration (MIC) for colistin in the range 1–64 μg/mL. Notably, 15 out of the 16 isolates demonstrated successful transfer of *mcr* genes via conjugation, indicative of their presence on plasmids. In contrast, the KK3 strain did not exhibit such transferability. Replicon types of *mcr-1*-containing plasmids included IncI2 and IncX4, while IncFIB, IncFII, and IncP1 contained *mcr-3*. Another single strain carried *mcr*-*1.1* on IncX4 and *mcr*-*3.5* on IncP1. Notably, one isolate contained *mcr-1.1* located on a chromosome and carrying *mcr*-*3.1* on the IncFIB plasmid. The chromosomal location of the *mcr* gene may ensure a steady spread of resistance in the absence of selective pressure. Retail meat products may act as critical reservoirs of plasmid-mediated colistin resistance that has been transmitted to humans.

## 1 Introduction

Antimicrobial resistance (AMR) is a serious global health issue that affects both humans and animals. Misuse and overuse of antibiotics in clinical treatment and with livestock contribute to the growth and spread of antimicrobial resistance bacteria (Liu et al., 2021). Colistin is a common antibiotic that has been utilized in the veterinary field for decades, not only for prophylaxis and treatment, but also for growth promotion. It is now regarded as a last resort against antimicrobial agents in therapy infections caused by multidrug-resistant, Gram-negative bacteria in humans (Zając et al., 2019). Inevitably, the increasing use of colistin in humans and animals has led to the emergence of colistin resistance in Gram-negative bacteria, with the rates of resistance continuing to increase (Kempf et al., 2016).

Normally, colistin resistance is thought to be chromosomally mediated (Meletis and Skoura, 2018). In 2016, a mobile colistin resistance (*mcr*) gene was identified in the IncI2-type plasmid of *E. coli* isolated from food animals and raw meat in China (Liu et al., 2016). The *mcr* gene encodes a membrane of phosphoethanolamine transferase that catalyzes the addition of phosphoethanolamine into lipid A to modify lipopolysaccharide (LPS), leading to a reduced negative charge on the outer membrane of Gram-negative bacteria (Vu Thi Ngoc et al., 2022). The *mcr* genes (*mcr-1* to *mcr*-*10*) have been successively reported globally; the *mcr-1* gene has been widely detected in animals, food products, humans and the general environment (Martiny et al., 2022). To date, the predominate plasmid carrying *mcr* has been reported in different plasmids based on their replicon types including IncX3, IncX4, IncI2, IncHI1, InHI2, IncK, IncP, IncF, IncFII, IncFIB, IncP, IncY, ColE10, and ColE10-like (Sun et al., 2018; Lu et al., 2020; Tang et al., 2022). Epidemiological studies have indicated that the spread of colistin-resistant *mcr*-carrying bacteria is not only a problem in hospitals, but also a major concern for environmental and food safety. In this regard, environmental sources, food-producing animals, foreign travel, and food trading have expedited the worldwide spread of *mcr* genes at the human-animal-environment interface (Liu and Song, 2019; Liu et al., 2021; Lopes et al., 2021; Nakano et al., 2021).

*Escherichia coli* (*E. coli*) is a commensal pathogen that causes gastrointestinal tract disease in humans and animals. This bacterium has been isolated at various sites in the food chain, typically usually due to fecal contamination (Macori et al., 2021). Often, it is used as an indicator organism to monitor AMR in livestock, food products, and humans (Singh et al., 2018; Pormohammad et al., 2019; Puangseree et al., 2022) and represents a major reservoir of antimicrobial resistance genes, mostly acquired through horizontal gene transfer (Puangseree et al., 2022). The spreading of *mcr*-carrying *E. coli* along the food chain is the most common transmission mode of colistin resistance, the *mcr-1* gene could have been disseminated to human populations through two primary pathways: the food supply chain and direct contact between animals and humans. Additionally, contamination of both freshwater and seawater systems likely played a role in the dissemination, subsequently leading to the contamination of vegetables and seafood (Chen et al., 2017; Lu et al., 2020). In Thailand, colistin-resistant *mcr*-carrying *E. coli* strains have been identified in humans ((Khine et al., 2020; Khanawapee et al., 2021; Paveenkittiporn et al., 2021; Trongjit and Chuanchuen, 2021; Nguyet et al., 2022; Phuadraksa et al., 2022). In our previous study, *mcr* genes were detected in 7.8% of slaughtered pork in Thailand (Khanawapee et al., 2021). Consequently, to comprehend the genomic characteristics in *mcr-*harboring *E. coli* strains in food animals in Thailand, we characterized the genetic features of *mcr-*harboring plasmids in *E. coli* that had been isolated from food animal products using whole-genome sequencing.

## 2 Materials and methods

### 2.1 Ethics statement

Ethical review and approval were not required because no human or animal specimens or data were used in the current study.

### 2.2 Bacterial isolates and identification

**Figure 1:**
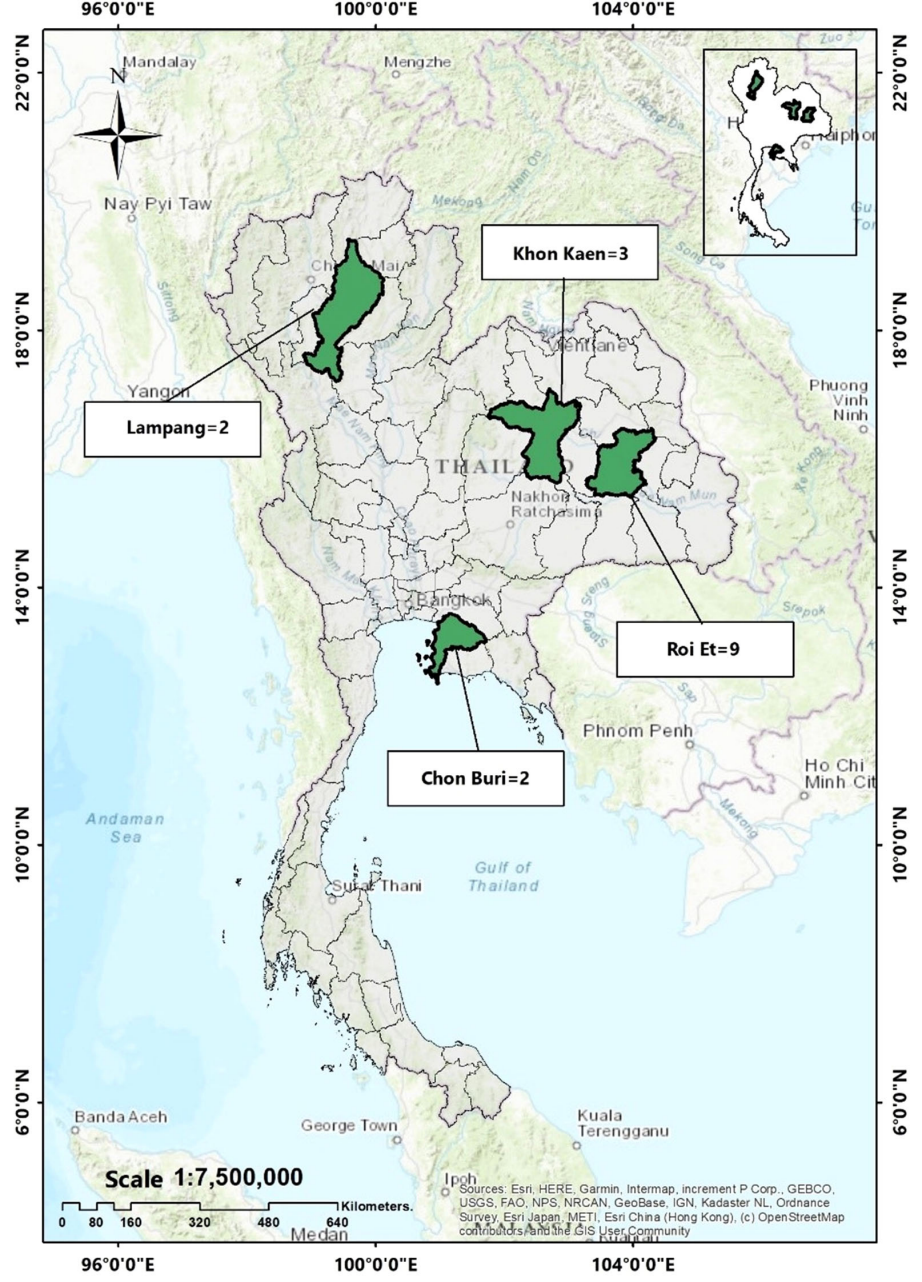
Geographical location of *mcr*-harboring *E. coli* in this study. (Khon Kaen = 3 isolates, Roi Et = 9 isolates, Lampang = 2 isolates and Chon Buri = 2 isolates).

In this study, a total of 16 *E. coli* isolates carrying *mcr* genes were analyzed. Ten of these isolates were collected from slaughtered pigs during 2014–2015, as reported in a previous study (Khanawapee et al., 2021). Furthermore, we identified *mcr* genes in six out of 40 *E. coli* isolates obtained from 63 retail meat samples collected between 2020–2021, (unpublished data) by using a polymerase chain reaction (PCR) methods as described elsewhere (Khanawapee et al., 2021). The geographical locations of sample collection are illustrated on the map in **Figure 1**.

### 2.3 Antimicrobial susceptibility testing

The six of *E. coli* isolates carrying *mcr* genes (P3.2, P8.5, P9.2, P9.3, P17.2 and C14.2) were subjected disk diffusion and broth microdilution (only colistin was performed with MIC) and interpreted according to the 2022 Clinical and Laboratory Standards Institute guidelines (Clinical and Laboratory Standards Institute (CLSI), 2022) subsequently, they were used for susceptibility testing of *mcr*-harboring *E. coli* isolates. *E. coli* ATCC 25922 was used as the control. While ten *E. coli* isolates carrying *mcr* genes have been reported in previous study (Khanawapee et al., 2021). The disk diffusion method was used to determine the susceptibility of clinical routine antimicrobial agents including ampicillin (AP; 10 µg), gentamicin (GM; 10 µg), amikacin (AK; 30 µg), amoxicillin/clavulanic acid (AUG; 30 µg), piperacillin-tazobactam (PTZ; 110 µg), cefepime (CPM; 30 µg), cefotaxime (CTX; 30 µg), ceftriaxone (CRO; 30 µg), ciprofloxacin (CIP; 5 µg), levofloxacin (LEV; 5 µg), ertapenem (ETP; 10 µg), imipenem (IMI; 10 µg), meropenem (MEM; 10 µg), ceftazidime (CAZ; 30 µg), chloramphenicol (C; 30 µg), tetracycline (T; 30 µg), fosfomycin (FOT; 200 µg), nitrofurantoin (NI; 300 µg), azithromycin (ATH; 5 µg), and trimethoprim (TM; 5µg). The MIC of colistin (colistin sulfate; Sigma-Aldrich, USA) were determined based on broth microdilution (Khanawapee et al., 2021). Multidrug-resistance status was assigned to isolates that were resistant to at least one agent from three or more different antimicrobial categories (Magiorakos et al., 2012).

### 2.4 Conjugation experiment

The transferability of *mcr* was assessed through a conjugation assay, utilizing *mcr-*harboring *E. coli* strains as donors and streptomycin-resistant *E. coli* UB1637 as a recipient. Briefly, both donor and recipient strains were culture in Luria-Bertani (LB) broth with shaking at 200 rpm at 37°C, then a 1:25 ration of donor to recipient strain was mixed in 1 ml of LB broth and incubated overnight at 37°C with gentle shaking 50 rpm. Transconjugants were selected on MacConkey agar plates containing streptomycin (3200 µg/mL) and colistin (4 µg/mL). The presence of *mcr* genes in transconjugants was detected via PCR method, and colistin MIC were determined to confirm the transconjugants as previously described (Phetburom et al., 2021).

### 2.5 Whole genome sequencing and bioinformatics analyses

Genomic DNA samples of the 16 *mcr-1* harboring *E. coli* strains were extracted using a MagMAX DNA Multi-Sample Ultra 2.0 Kit (Appliedbiosystems by Thermo Fisher Scientific; Vilnius, Lithuania). DNA concentrations were determined using a Qubit dsDNA HS assay kit (Thermo Fisher Scientific; Waltham, MA, USA). Whole-genome sequencing was performed using the HiSeq 3000 (Illumina; CA, USA) and MinION (Oxford Nanopore Technologies; Oxford, UK) platforms. The DNA library was generated using the NEBNex Ultra II DNA Library Prep Kit for Illumina (New England Biolabs; MA, USA) according to the manufacturer. The Illumina HiSeq platform was prepared with 150-bp paired-end reads. We applied the Fastp v0.19.5 program (Chen et al., 2018) with default parameters for quality filtering of the Illumina reads and adapter trimming for the sequences using the Skewer v0.2.2 program (Jiang et al., 2014). The FastQC v0.118 program^1^ was used for quality checking of the Illumina reads.

Library preparation for ONT sequencing followed the rapid barcoding DNA sequencing protocol using the SQK-RBK004 kit (Oxford Nanopore Technologies; Oxford, UK), and the libraries were sequenced using a single R9 version/FLO-MIN106D flow cell on a MinION Mk1B sequencer (Oxford Nanopore Technologies; Oxford, UK). We base called and demultiplexed the raw data using the Guppy v5.0.11 (ONT) program. The ONT adapters were trimmed using the Porechop v0.2.4 program^2^ (Wick et al., 2019). Quality control of the ONT reads was undertaken using the NanoPlot v1.28.1 program^3^ (De Coster and Rademakers, 2023). Hybrid assemblies with the ONT and Illumina data were performed using Unicycler v0.48 (Wick et al., 2017) and the genome sequences were checked for quality using the QUAST v5.0.2 program (Gurevich et al., 2013). The genome sequences were processed using the DDBJ Fast Annotation and Submission tool (Tanizawa et al., 2018) for annotation. Default parameters were used for all software programs unless otherwise specified.

Genomes were *in silico* typed using the following databases: sequence types (MLST 2.0) (Larsen et al., 2012), serotypes (SerotypeFinder 2.0) (Joensen et al., 2015), subtypes (CHtyper 1.0) (Camacho et al., 2009), while FimH types were identified using the FimTyper 1.0 program (Roer et al., 2017). Plasmid replicon types were identified using the PlasmidFinder 2.1 program (Carattoli et al., 2014) from the Center for Genomic Epidemiology (CGE). Clermont phylotyping^4^ was performed. Acquired resistance genes and chromosomal mutation-mediated antimicrobial resistance genes were identified using the ResFinder 4.1 program (Zankari et al., 2012) from CGE and the resistance gene identifier from the Comprehensive Antibiotic Resistance Database (Alcock et al., 2023). A SNP-based phylogenetic tree was constructed to compare twelve *E. coli* strains isolated from patients in Thailand (Paveenkittiporn et al., 2021; Boueroy et al., 2022) (**Supplementary Table 1**) with the sixteen *mcr-1* harboring *E. coli* strains from food-animal products in this study. The construction of the tree was carried out using REALPHY online tool (Bertels et al., 2014). Phylogenetic analysis of *mcr* carrying plasmids was applied using Mashtree (Katz et al., 2019) and visualized using the Interactive Tree of Life (iTOL) tool (Letunic and Bork, 2021). The Easyfig software (version 2.2.5) was used for the comparative and visualize the region of plasmids carrying *mcr* genes. (Sullivan et al., 2011). The sequence similarity search was performed using BLAST against the NCBI nucleotide database. In order to examine the distribution and relationships of other plasmid carrying *mcr*, plasmid sequences were downloaded from NCBI and compared (**Supplementary Table 2**).

### 2.6 Data availability statement

The complete genomes were deposited in GenBank under Bioproject Accession No. PRJNA525849.

## 3 Results

### 3.1 Antimicrobial susceptibility of *mcr*-carrying *E. coli* strains

In total, of the 16 *E. coli* carrying *mcr* isolates, 11 contained *mcr-1, 3* isolates harbored *mcr-3*, and 2 isolates contained *mcr*-*1* and *mcr*-*3.* The antimicrobial susceptibility results are shown in **Table 1**. All strains were defined as multidrug resistance (MDR) based on resistance to at least three classes of antibiotics (Magiorakos et al., 2012). Of the 16 isolates, 15 (93.75%) were resistant to ampicillin, 12 (75%) were resistant to trimethoprim and tetracycline, 11 (68.75%) were resistant to chloramphenicol, and 10 (62.5%) to ciprofloxacin, whereas there was no resistance to fosfomycin and carbapenem was detected. The colistin MIC of these isolates was in the range 1–16 µg/mL, as shown in **Table 1**. We detected only one isolate (RE46) did not resist to colistin (MIC = 1 ug/ml), whereas the rest isolates were resistant. We detected only one isolate (RE46) did not show resistance to colistin (MIC = 1 ug/ml), whereas the remaining isolates were resistant.

**Table 1:** Antimicrobial susceptibility profiles of *mcr-*harboring *E. coli* isolates.

Antimicrobial Family	Isolate Antibiotics	P3.2	P8.5	P9.2	P9.3	C14.2	P17.2	RE46	LP71	LP72	KK3	KK76	KK79	RE40	CB70	RE14	CB62	No. Resistant (%)
Penicilins	Ampicillin (AP)	**R**	**R**	**R**	**R**	**R**	**R**	**R**	**R**	**R**	**R**	S	**R**	**R**	**R**	**R**	**R**	15 (93.75%)
Cephems	Ceftriaxone (CRO)	S	S	**R**	S	S	**R**	S	**R**	S	S	S	S	S	S	S	**R**	4 (25%)
	cefotaxime (CTX)	S	S	**R**	S	S	**R**	S	**R**	S	S	S	S	S	S	S	**R**	4 (25%)
	Ceftazidime (CAZ)	S	S	**R**	S	S	S	S	**R**	S	S	S	S	S	S	S	**R**	3 (18.75%)
	Cefepime (CPM)	S	S	**R**	S	S	S	S	**R**	S	S	S	S	S	S	S	**R**	3 (18.75%)
Carbapenems	Meropenem (MEM)	S	S	S	S	S	S	S	S	S	S	S	S	S	S	S	S	0 (0%)
	Imipenem (IMI)	S	S	S	S	S	S	S	S	S	S	S	S	S	S	S	S	0 (0%)
	Ertapenem (ETP)	S	S	S	S	S	S	S	S	S	S	S	S	S	S	S	S	0 (0%)
Phenicols	chloramphenicol (C)	S	**R**	**R**	**R**	S	S	**R**	S	**R**	**R**	**R**	**R**	**R**		**R**	**R**	11 (68.75%)
Fluoroquinolones	Ciprofloxacin (CIP)	S	S	**R**	S	**R**	**R**	**R**	**R**	S	**R**	S	S	**R**	**R**	**R**	**R**	10 (62.5%)
	Levofloxacin (LEV)	S	S	**R**	S	**R**	**R**	S	S	S	S	S	S	S		S	**R**	4 (25%)
Aminoglycosides	Gentamicin (GM)	S	S	**R**	S	S	**R**	S	**R**	S	S	S	S	S	**R**	**R**	**R**	6 (37.5%)
	Amikacin (AK)	S	S	S	S	S	S	S	S	S	S	S	S	S	**R**	S	S	1 (6.25%)
Tetracyclines	Tetracycline (T)	**R**	**R**	**R**	**R**	**R**	S	**R**	S	**R**	S	**R**	**R**	**R**	S	**R**	**R**	12 (75%)
Macrolides	Azithromycin (ATH)	S	S	**R**	S	S	**R**	S	S	**R**	**R**	S	S	S	S	S	**R**	5 (31.25%)
Fosfomycin	Fosfomycin (FOT)	S	S	S	S	S	S	S	S	S	S	S	S	S	S	S		0 (0%)
Nitorfurans	Nitrofurantoin (NI)	S	S	S	S	S	**R**	S	S	S	S	S	S	S	S	S	**R**	2 (12.5%)
Sulfonamides	Trimethoprim (TM)	S	**R**	**R**	S	**R**	**R**	**R**	S	**R**	S	**R**	**R**	**R**	**R**	**R**	**R**	12 (75%)
β-lactam + β-lactamase inhibitor	Piperacillin- Tazobactam (PTZ)	S	**R**	**R**	**R**	**R**	S	S	S	S	S	S	S	S	S	S	S	4 (25%)
	Amoxicillin/clavulanic acid (AUG)	S	S	S	S	S	**R**	S	S	S	S	S	S	**R**	**R**	S	S	3 (18.75%)
Lipopeptides	MIC colistin (μg/ml)	8	8	8	8	16	8	1	4	4	4	4	4	4	8	4	4	

S, Susceptible; R, Resistant (marked in bold); Results interpreted based on CLSI 2022.

### 3.2 Conjugation

Of the 16 *mcr* harboring *E. coli* isolates, 15 successfully transferred the colistin resistant gene (*mcr*) by conjugation to the recipient strain (*E. coli* UB1637), with the KK3 strain being the exception. All transconjugants had harboring *mcr-1* and *mcr-3* depend on donor and colistin MIC values in the range 8–16 µg/mL, suggesting the transferred *mcr* genes were located on conjugative plasmids.

### 3.3 Genotypic profile of *mcr*- harboring *E. coli* isolates

Whole-genome sequencing (WGS) of the *mcr*-harboring *E. coli* (**Supplementary Table 3**) including Clermont phylogroup, sequence types (ST), serotype, CHType, FimH type, plasmids replicon types, antibiotic resistance and virulence genes was shown in **Table 2**. The Clermont phylotyping identified 7 isolates (43.75%, 7/16) belonging to group A, 3 to group D, and 2 for each of groups C, B1, and B2 (**Table 2**). FimH398 was detected in 4 isolates (25%, 4/16). Two isolates had FimH54 and FimH23, while the remaining isolates belonged to individual FimH types. For the CHType, 27-398 was found in 2 isolates, with different CHType represented by the remaining isolates. The MLST revealed 13 STs among these isolates (**Table 2**). ST10 was found in 3 isolates, with 2 isolates belong to ST1114 and 11 STs.

**Table 2:** Genomic features *of mcr-1* and *mcr-3* carrying *E. coli*.

Strain	Year of isolation	Accession	Serotype	Phylo group	CHType	ST	FimH type	Location of *mcr* gene	Other plasmid replicons	Acquired resistances genes	Virulence genes	Mutation gyrA & parC
P3.2	2021	JAVHZS000000000	O126:H2	A	11-54	10	*fimH54*	*mcr-1.1*/IncX4	IncFII(pCoo), p0111	*bla*TEM-1B, *tet(A)*	*astA, csgA, fimH, gad, hlyE, cba, cma, etsC, iss, terC, traJ, tsh, yehA, yehB, yehC, yehD*	
P8.5	2021	JAVHZR000000000	H1 2	C	11-0	218	–	*mcr-1.1*/IncX4	IncFIA(HI1), IncFIB(K), IncY	*bla*TEM-1B, *aadA1, aadA2, mef(B), dfrA12, sul3, tet(A), tet(M), cmlA1*	*csgA, gad, nlpl, terC, yehA, yehB, yehC, yehD*	
								*mcr-3.5/*IncP1				
P9.2	2021	JAVHZQ000000000	O68:H30	C	11-24	34	*fimH24*	*mcr-1.1*/IncX4	IncFII, IncHI2, IncHI2A, IncN	*bla*TEM-1B, *bla*CTX-M-14, *bla*CTX-M55, *aac(3)-IId, aadA22, aph(6)-Id, erm(42), qnrS1, sul2, tet(A), tet(X4), floR*	*csgA, fimH, gad, hlyE, terC, nlpl, traT, traJ, yehA*	gyrA:p.S83L gyrA:p.D87H parC:p.S80I
P9.3	2021	JAVHZP000000000	O155:H5	A	11-760	7589	*fimH760*	*mcr-1.1*/IncX4	IncR, IncX1	*bla*TEM-1B, *qnrS1, sul2, tet(A), tet(M), tet(X4), floR*	*csgA, fimH, tia, etsC, gad, hra, terC, traT, traJ, nlpl, yehA, yehB, yehD*	
C14.2	2021	JAVHZO000000000	O7:H18	D	26-54	12817	*fimH54*	*mcr-1.1*/IncX4	IncFIB(pB171), IncFII, IncY	*bla*TEM-1B, *aadA1, qnrS13, dfrA1, tet(A)*	*air, astA, fimH, chuA, cma, cvaC, eilA, AslA, hlyF, gad, iha, iroN, iss, kpsE, kpsMII_K5, ompT, papC, sitA, terC, traT, traJ, nlpl*	gyrA:p.S83L parC:p.S80R
P17.2	2021	JAVHZN000000000	O2:H26	A	27-398	1114	*fimH398*	*mcr-1.1*/IncX4	IncFIB(AP001918), IncY	*bla*TEM-1B, blaCTX-M-14, blaCTX-M55, *aac(3)-IId, aph(3’’)-Ib, aph(6)-Id, bleO, erm(B), mph(A), qnrS1, OqxA, OqxB, tet(A), tet(M)*	*astA, csgA, fimH, gad, kpsE, kpsMII, terC, traT, traJ, nlpl, yehA*	gyrA:p.S83L gyrA:p.D87Y parC:p.S80I
RE46	2014	JAVHZM000000000	O32:H19	A	19-32	5506	*fimH32*	*mcr-1.1*/IncX4	IncFII(pCoo)	*bla*TEM-1B, *aadA16, aph(3’’)-Ib, aph(6)-Id, dfrA27, sul2, tet(A), floR*	*csgA, fimH, gad, hra, iss, ipfA, papC, tia, papA_F19, hlyE, sitA, terC, traT, traJ, nlpl, yehA, yehC, yehD*	
LP71	2014	JAVHZL000000000	H5	B1	11-398	7463	*fimH398*	*mcr-1.1*/IncI2	IncFIA(HI1), IncHI1A, IncHI1B(R27), IncR	*bla*TEM-B, *aadA1, aph(3’’)-Ib, mef(B), dfrA12, sul3, tet(A), tet(M), cmlA1, lnu(F)*	*csgA, fimH, gad, terC, nlpli, traT, traJ, yehA, yehB, yehD*	gyrA:p.S83L
LP72	2014	JAVHZK000000000	O184:H31	B2	41-86	101	*fim*H86	*mcr-1.1*/IncI2	IncFIB(AP001918)	–	*csgA, fimH, gad, cma, hlyF, cvaC, hlyE, iroN, iss, tsh ipfA, mchF, ompT, sitA, terC, yehA, yehC, yehD*	
KK3	2014	JAVHZJ000000000	H32	D	11-41	10	*fimH41*	*mcr-1.1* Chromosome	IncFIA(HI1), IncFIB(K)	*bla*TEM-1B, *aadA1, aadA2b, aadA24, mef(B), qnrS1, sul3, lnu(F)*	*csgA, fimH, gad, terC, nlpl, yehD*	
KK76	2014	JAVHZI000000000	H45	B2	4-32	155	*fimH32*	*mcr-1.1*/IncI2	IncFIB(pLF82-PhagePlasmid), IncR	*aadA1, aadA2, dfrA12, sul3, tet(A), tet(M), cmlA1*	*csgA, fimH, astA, gad, hlyE, ipfA, nlpl, terC, traT traJ, yehC, yehD*	
KK79	2014	JAVHZH000000000	O50:H32	D	11-23	10	***fimH*23**	*mcr-1.1*/IncX4	IncFIB(AP001918), IncR, IncX1, IncY	*bla*TEM, *aadA1, aadA2, dfrA12, sul3, tet(A), tet(M), cmlA1, floR*	*csgA, fimH, gad, hra, iss, tia, nlpl, terC, traT, tsh, traJ, yehA, yehB, yehD*	
RE40	2014	JAVLUQ000000000	O172:H23	A	4-31	7625	*fimH31*	*mcr-3.1/*IncFIB(AP001918)	IncFIB(H89-PhagePlasmid), IncI1-I(Alpha), IncN, IncQ1	*bla*CTX-M-55, *aac(3)-IId, aph(3’’)-Ib, aph(6)-Id, qnrS1, tet(A), tet(M), catA2*	*csgA, fimH, hlyE, gad, ipfA, nlpl, terC, yehB, yehD*	gyrA:p.S83L gyrA:p.D87N parC:p.S80I
CB70	2014	JAVHZG000000000	O174:H2	A	11-946	4015	*fimH946*	*mcr-1.1*/Chromosome	IncFIB(K), IncX1, IncY	*bla*TEM-1B, *aac(3)-IId, aph(3’’)-Ib, aph(6)-Id, qnrS1, dfrA14, sul2, tet(A), tet(M)*	*astA, csgA, fimH, gad, terC, traT traJ, yehA, yehB*	
								*mcr-3.1*/IncFIB(AP001918)				
RE14	2014	JAVLUP000000000	O41:H26	B1	11-23	5995	*fimH23*	*mcr-3.5*/IncP1	IncFIA(HI1), IncFIB(H89-PhagePlasmid), IncHI1A, IncHI1B(R27), IncN	*bla*TEM-1B, *aadA1, aac(3)-IId, aph(3’’)-Ib, aph(6)-Id, mef(B), qnrS1, dfrA12, sul3, tet(B), cmlA1, lnu(F)*	*csgA, fimH, gad, terC, nlpl, yehA, yehB, yehD*	
CB62	2014	JAVHZF000000000	O33:H26	A	27-398	1114	*fimH398*	*mcr-3.1*/IncFII, IncR	IncY	*bla*TEM-1B, blaCTX-M-55, *aac(3)-IId, aadA1, aadA2, mdf(A), qnrS1, dfrA12, sul3, tet(A), tet(M), catA2, cmlA1*	*astA, csgA, fimH, gad, aslA, terC, nlpl, traT, traJ, yehD*	gyrA:p.S83L

**Figure 2:**
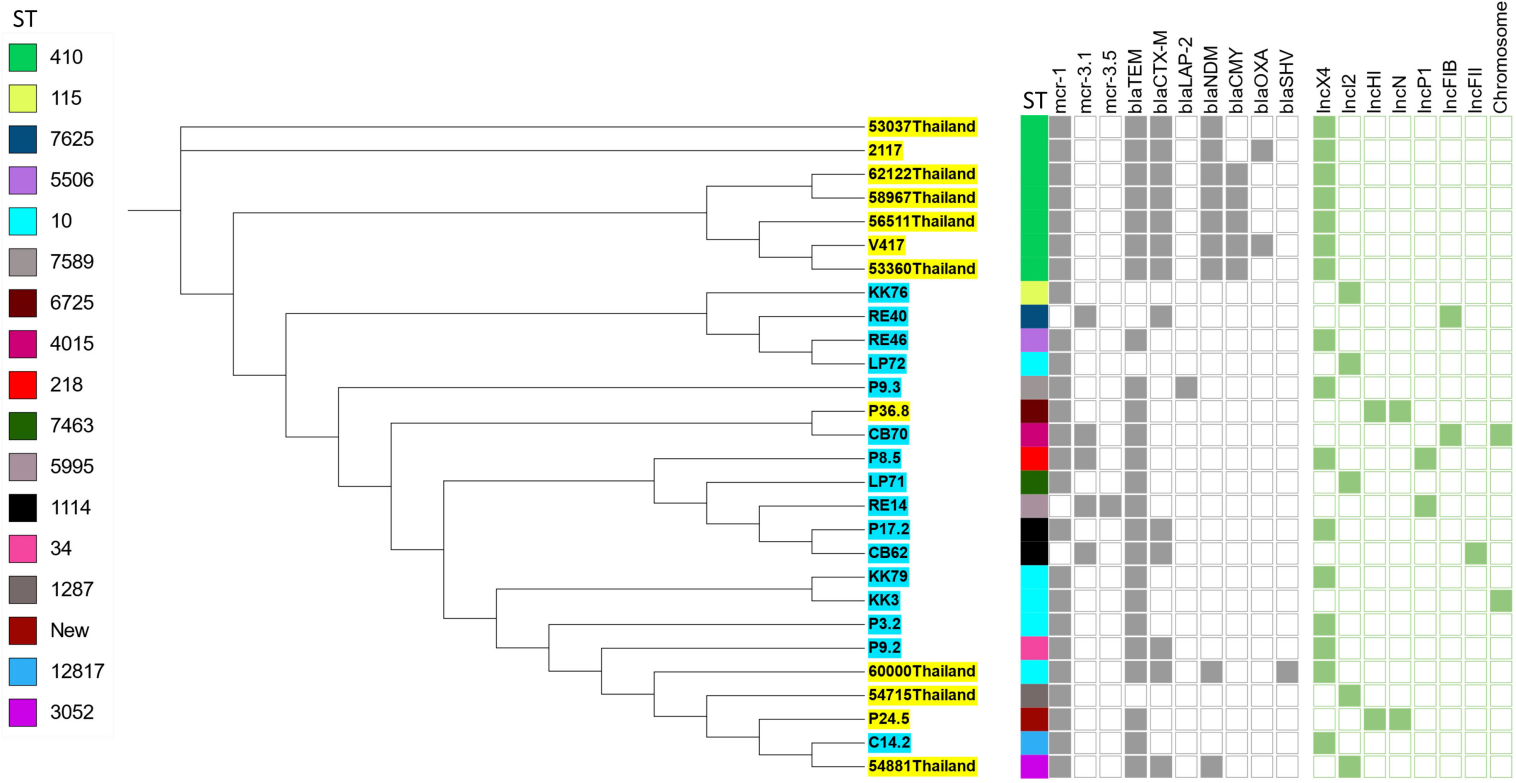
Phylogenetic tree of *mcr-*harboring *E. coli*, where blue color represents isolates from meat (pork and chicken) in this study, while yellow represents isolates from human specimens.

To analyze the genetic relationship of the *mcr*-carrying *E*. *coli* isolated in this study, we conducted a phylogenomic analysis using available genomes of *mcr*-carrying *E. coli* (n = 12) isolated from patients in Thailand in another study (Boueroy et al., 2022). The phylogenetic tree had two main distinct clades. The *mcr-*carrying *E. coli* isolates from our study had a related genetic background in a cluster, except for C14.2 that was closely related to Thai human stains 54881, 54715, and 60000 (**Figure 2**). The strains KK76, RE40, RE46, and LP72 were located on the same cluster as the Thai human strains 53037, 2117, 62122, 58967, 56511, 53360, and V417 (**Figure 2**).

The genomic analysis of *mcr-*harboring *E. coli* revealed a high number of resistance genes, with up to 37 different antibiotic resistance genes that conferred resistance to 8 antibiotic classes, consisting of β-lactam resistance genes, aminoglycoside, tetracycline, macrolide, phenicol, fluoroquinolones, folate pathway antagonist, and sulfonamide genes. Additionally, mutations in the *gyr*A (S83L, D87H, D87Y, D87N) and *par*C (S80I, S80R) genes were identified in the C14.2, LP71, CB62, P9.2, P17.2, and RE40 strains, as displayed in **Table 2**, which confer high-level resistance to fluoroquinolones.

A total of 35 distinct virulence genes were identified within genomes of *mcr* positive *E. coli* isolates. All 16 *mcr* containing *E. coli* isolates carried the virulence genes *gad* (glutamate decarboxylase), *ter*C (tellurium ion resistance protein, and *csg*A (curlin major subunit). Furthermore, 15 *mcr* bearing *E. coli* isolates carried *fim*H (Type 1 fimbriae), 14 *mcr* bearing *E. coli* isolates carried nlpl (lipoprotein NlpI precursor), and 11 *mcr* bearing *E. coli* isolates carried *tra*J (Protein TraJ (a positive regulator of conjugal transfer operon)) and *yeh*A (outer membrane lipoprotein, YHD fimbriael cluster), as shown in **Table 2**.

### 3.4 Comparison of plasmids containing *mcr* in *E. coli*

The WGS analysis of the 16 *mcr*-harboring *E. coli* isolates revealed that 11 *mcr-1* genes and 4 *mcr-3* genes were located on the plasmids; however, the 2 *mcr*-*1* genes (KK3 and CB70 strains) were located on the chromosome (**Table 2**). Among the 16 *mcr*-positive isolates identified, two strains harbored both *mcr-1* and *mcr-3* genes (CB70 and P8.5; **Table 2**). Seven isolates contained *mcr-1.1* genes were located on IncX4 plasmids, while 3 isolates harbored *mcr-1.1* genes on IncI2 plasmids. The *mcr-3* genes were detected on IncFIB (n=2), IncP1 (n=2), and IncFII (n=1) plasmids. Notably, strain KK3 exhibited chromosomal carriage of *mcr-1.1*, while strain CB70 harbored *mcr-1.1* on its chromosome and *mcr-3.1* on an IncFIB plasmid (**Table 2**).

**Figure 3:**
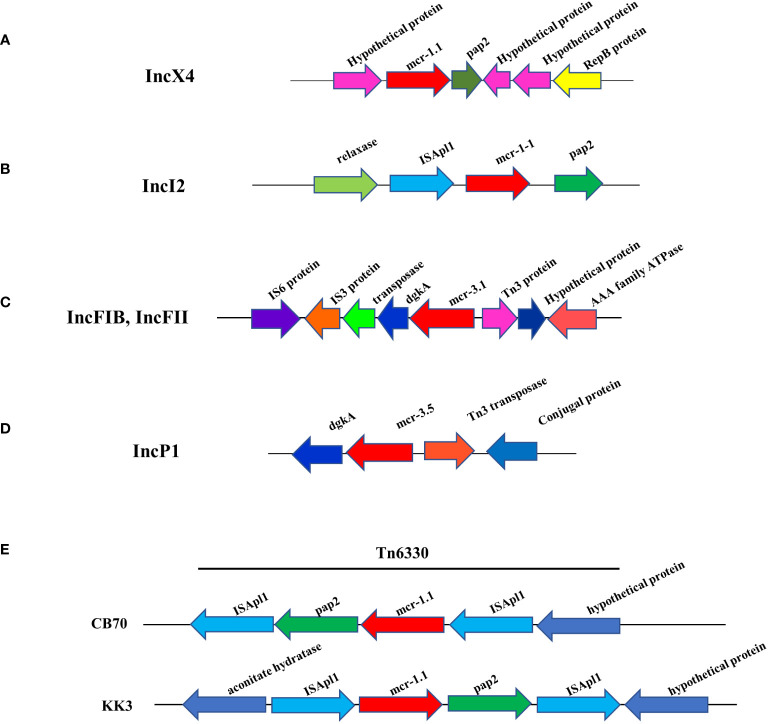
Schematic partial representation of genetic surrounding of *mcr-*harboring plasmid replicon types of *E. coli*, where blue color represents isolates from meat (pork and chicken) in this study and yellow represents isolates from human specimens. **(A)** IncX4 containing *mcr-1.1*, **(B)** IncI2 containing *mcr-1.1*, **(C)** IncF containing *mcr-1.1*, **(D)** IncP1 containing *mcr-1.1 *and **(E)** showing *mcr-1.1* located on a chromosome.

Analysis of genetic context of the 8 plasmid IncX4 samples containing *mcr-1* showed the conserved *mcr*-*1.1*-*pap*2 segment (**Figure 3A**), a putative pap2 protein, a pap2 membrane-associated lipid phosphatase, which have transmembrane domains and are involved in the phosphatidic acid pathway and lipid metabolism and signal (Fan et al., 2014; Wang et al., 2018; Gaballa et al., 2023). No other antibiotic resistance genes were detected in the whole IncX4 plasmid, while 3 *mcr*-*1.1* were carried on the IncI2 plasmid that revealed the genetic environment of *mcr-1* to be relaxase-IS*Apl*1- *mcr*-*1.1*-*pap*2 (**Figure 3B**). Furthermore, 3 *mcr-3.1* genes showed arrangements of the IS3 family transposase-transposase-*dgk*A-*mcr*-*3.1*-*Tn*3family transposase (**Figure 3C**). The plasmids carried *mcr-3.5* containing diacylglycerol kinase (*dgk*A) downstream and *Tn*3 transposase and a conjugal protein upstream (**Figure 3D**). Notably, the genetic features of *mcr-1.1* on the chromosomes of CB70 and P8.5 contained IS*Apl*1-*pap*2-*mcr*-*1.1*-IS*Apl*1 (**Figure 3E**).

**Figure 4:**
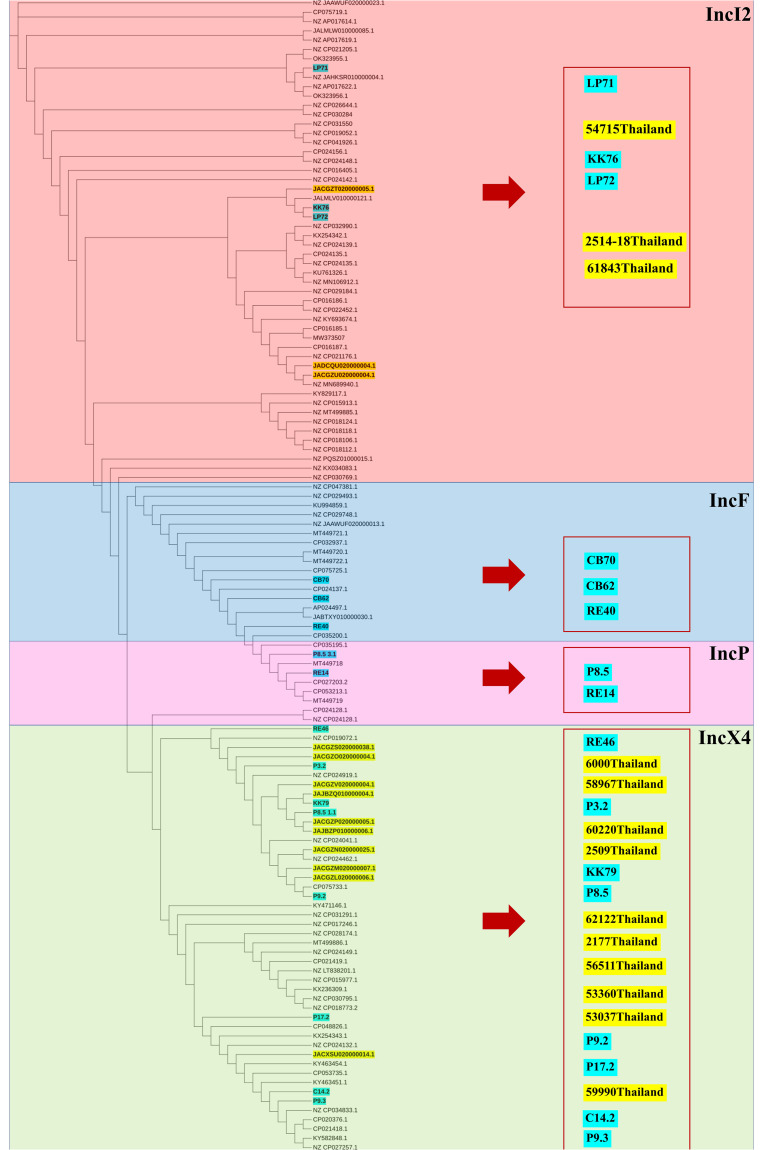
Phylogenetic tree of *mcr-*harboring plasmid replicon types of *E. coli*, where blue color represents isolates from meat (pork and chicken) in this study and yellow represents isolates from human specimens.

**Figure 5:**
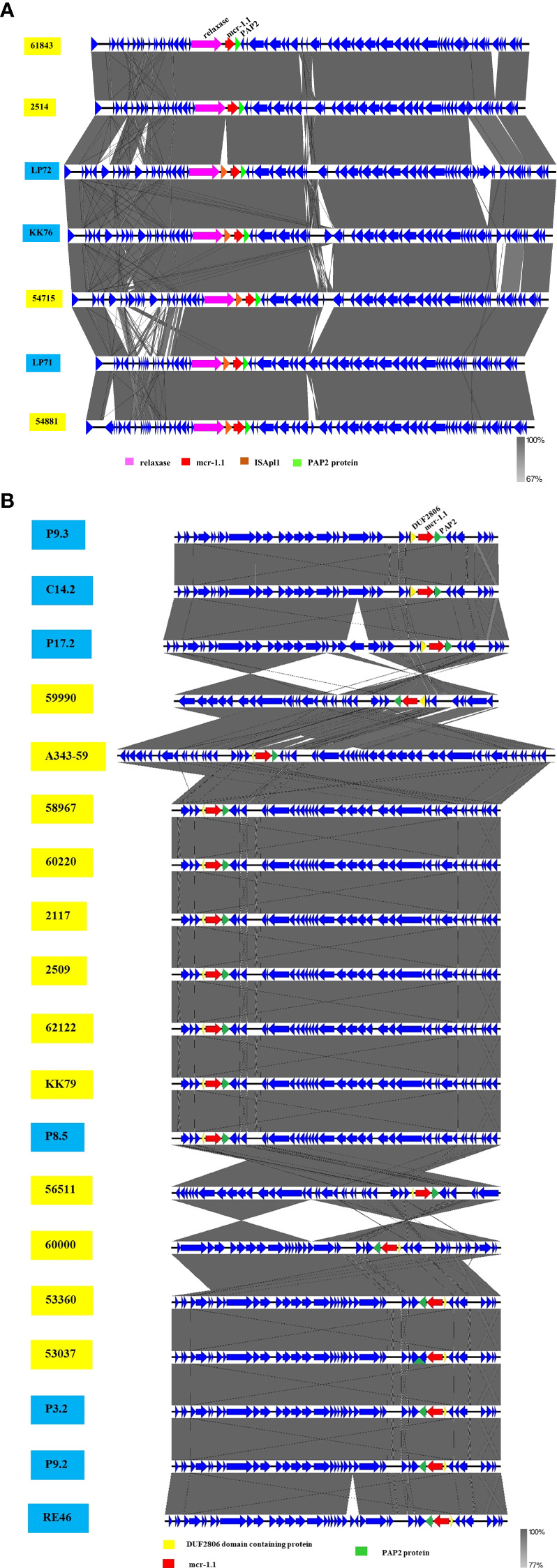
Schematic representation of sequence alignment analysis of IncI2 **(A)** and IncX4 **(B)** plasmids carrying *mcr-1.1*, comparing meat and human specimens. Boxed arrows represent position and transcriptional direction of ORFs, with mobile genetic elements (*mcr-1.1*) in red, pap2 protein in green, relaxase protein in pink, ISApl1 in orange, and other genes or hypothetical ones in blue. The ID strain in light blue box represents isolates from meat (pork and chicken) in this study and yellow box represents isolates from human specimens.

The phylogenetic relationships of the plasmid replicon type IncX4 and IncI2 carrying *mcr*-*1* in *E. coli* in our study were closely related with the plasmids IncX4 and IncI2 harboring *mcr*-*1* human *E. coli*. (**Figure 4**). The comparative analysis of the IncI2 and IncX4 types carrying *mcr-1* in *E. coli* showed that both the human and meat strains were similar and contained *mcr*-*1.1-pap*2. (**Figure 5**).

## 4 Discussion

There have been reports of the prevalence of plasmid-mediated colistin resistance in *E. coli* in a wide range of Enterobacterales, especially in *E. coli* from various sources, environments (Lopes et al., 2021; Mohsin et al., 2021; Nakano et al., 2021) food-producing animals (Huang et al., 2017), livestock (Migura-Garcia et al., 2020), and humans (Li B. et al., 2018; Yamaguchi et al., 2020). Other studies have shown that the plasmid type is important in *mcr* dissemination due to horizontal gene transfer in various sources. In Thailand, the *mcr* gene has been identified in clinical samples (Paveenkittiporn et al., 2021; Boueroy et al., 2022) and animals (Khine et al., 2020; Khanawapee et al., 2021; Trongjit and Chuanchuen, 2021). Here, we characterized the genetic feature and accessory gene of 16 *mcr-1* and *mcr-3* carrying *E. coli* strains isolated from meat samples in the food supply chain. Almost all isolates exhibited resistance to colistin, however, only one isolate did not resist. This result is consistent with other studies that reported colistin MIC values below 2 µg/ml but present *mcr* gene ((Khanawapee et al., 2021; Pungpian et al., 2021).

In recent decades, the incidence of extended-spectrum β-lactamases (ESBL)-producing *E. coli*, contributing to a growing incidence of community-acquired extra-intestinal infections in humans, animals and wide range of food animals product, including beef, pork and chicken meat globally (Shafiq et al., 2019; Shafiq et al., 2021; Shafiq et al., 2022). Our study, *E. coli* harboring *mcr-1* and *mcr-3* were detected together with ESBL genes (*bla*_TEM-1B_ *, bla*_CTX-M-14_ and *bla*_CTX-M-55_). Among them, *bla*_TEM-1B_ was the most common found in thirteen *mcr* positive *E. coli*, these results correlated with previous findings in Thailand that demonstrated the concurrent carriage of *mcr-1.1* with *bla*_CTX-M-14_, *bla*_CTX-M-55_, and *bla*_TEM-1B_ (Leangapichart et al., 2023). Moreover, the coexistence of *mcr* with ESBL genes in *E.coli* was report from other studies such as China and Pakistan, *E. coli* co-harboring *mcr* with ESBL genes such as *bla*_CTX-M-14_, *bla*_CTX-M-55_, and *bla*_TEM-1B_ (Shafiq et al., 2019; Shafiq et al., 2022).

Our study, identified 12 different STs, containing 3 ST10 strains and 1 ST34 strain, which could be considered as a broad range of hosts to carry *mcr* that are found in humans, animals, vegetables, and wastewater (Shen et al., 2020) that have been reported in humans and animals in Thailand (Khanawapee et al., 2021; Paveenkittiporn et al., 2021; Boueroy et al., 2022). These STs were most common among the *mcr-1* positive isolates (Li et al., 2022). The majority of phylogroups in our studies belonged to group A (43.75%), similar with other reports (Bosák et al., 2019; García-Meniño et al., 2019).

Horizontal gene transfer (HGT) via plasmids plays a critical role in the dissemination of antibiotic resistance determinants among Gram-negative bacteria (Sun et al., 2017). Notably, the *mcr* genes, responsible for colistin resistance, are located on diverse plasmids, encompassing more than 20 incompatibility groups identified as carriers of *mcr-1* (Liu et al., 2023). The IncX4, IncH2 and IncI2 replicon represent the majority, exceeding 90% of plasmids that have been identified globally. While IncX4 was the predominant plasmid group carrying the *mcr-1* gene in Europe and IncI2 in Asia, recently indicates a global shift towards IncX4 prevalence (Sismova et al., 2023). Our analysis of *mcr* carrying plasmid sequences, revealing a significant predominance of *mcr-1* on IncX4 replicons, with additional presence on IncI2, IncP1, IncFIB and IncFII plasmids. These finding resonate with our previously published data, demonstrating *mcr-1* carriage by IncX4 and IncI2 replicon in *E. coli* in humans in Thailand (Boueroy et al., 2022). This supports the notion of *mcr* gene emergence within specific plasmid groups in Enterobacterales from various reservoirs, including animals, food, environment, and humans (Boonyasiri et al., 2023; Leangapichart et al., 2023). Additionally, our experiments revealed the conjugative transfer capacity of IncX4 plasmids harboring *mcr-1*. Although this observation is limited to our laboratory conditions, it may offer a partial explanation for the increasing dominance of IncX4 in *mcr-1* dissemination. Its high self-transferable at high frequencies (Sun et al., 2017; Bai et al., 2018). The high efficiency of horizontal transfer via this plasmid family poses a significant risk, considering that colistin is considered a last-line antibiotic for the treating life-threatening infections in humans (Sismova et al., 2023).

The *pap*2 superfamily protein was detected upstream but was not detected in the insertion sequence IS*Apl*1 of any of the *mcr*-*1-* bearing InX4 plasmids, which indicated that the *mcr*-1 located in IncX4 was stable.

The genetic context analysis surrounding the *mcr* genes in our studies was hampered by the inherent ambiguity of the conjugation assay for determining the origin of transferred genes. The conjugation assay inability to definitively differentiate between plasmid and chromosomal transfer could lead to misinterpretations. This ambiguity stems from two scenarios. Firstly, *mcr* genes may reside on plasmids that integrated into the chromosome prior to conjugation. In such instances, the transferred genes would appear chromosomal despite their initial plasmid origin (Rafii and Crawford, 1988; Willett, 1988).

Furthermore, the *ISAPl*1 insertion sequence has been implicated as a crucial element facilitating the translocation of *mcr*-*1* into diverse plasmid types. This sequence has been localized on the chromosome through the formation of circular intermediates (Trongjit and Chuanchuen, 2021). The close genetic association between *ISApl*1 and *mcr-1* suggests that *ISApl*1 may play a pivotal role in the dissemination of *mcr-1* (Li et al., 2017). The three isolates of *mcr-1* located on the IncI2 plasmid contained IS*APl*1-*mcr*-*1*-*pap*2, which is one pattern of the transposon *Tn*6330 (Snesrud et al., 2018). In our study, the *mcr*-*3.1* gene was located on the IncFIB and IncFII plasmids, while *mcr*-3.5 was located on the IncP1 plasmid, to a study in China (Li J. et al., 2018). Both plasmids contained a diacylglycerol kinase (*dgkA*) upstream and this genomic context similar to another study (Phuadraksa et al., 2022). In addition, almost all the *mcr*-carrying plasmids could transfer to the recipient *E. coli* strain, which was in agreement with another study that concluded these plasmids were conjugative and dissemination could easily occur (Trongjit and Chuanchuen, 2021).

Other reports noted that the presence of the chromosomally encoded *mcr* gene is very rare (3.5–4%) (Li R. et al., 2018; Shen et al., 2020); however, a recent study reported *E. coli* strains harboring chromosomally encoded *mcr-1* were found in 26-37% isolates from humans and animals in Vietnam (Vu Thi Ngoc et al., 2022). Recently, co-harboring of *mcr*-*2* and *mcr-3* on chromosomes in *E. coli* isolated from a healthy human was documented in Thailand (Phuadraksa et al., 2022). The current study revealed 2 *mcr*-*1* harboring *E. coli* strains (KK3 and CB70) contained the segment of transposase IS*Apl*1-*mcr*-*1-pap*2-*ISAp*l1 (*Tn*6330) in the chromosome that would increase its stability and be highly mobilizable in spreading *mcr* genes due to vertical transfer.

## 5 Conclusion

Retail meat products (primarily pork and chicken), as a main food type and an important component of the food chain, were contaminated by *mcr* genes. Such meat products may serve as reservoirs of this antimicrobial-resistant gene that can affect humans. This finding supports the potential for the distribution and transmission of the colistin-resistant *mcr* gene is mediated by plasmids. Therefore, the *mcr* gene can spread via food chains, with the source of the strains suggesting that *mcr-1* may be a public health risk by spreading to humans through farm to fork processes. Therefore, ongoing monitoring and investigations of *mcr* genes in agricultural sector are required to control and prevent their spread.

## Data availability statement

The datasets presented in this study can be found in online repositories. The names of the repository/repositories and accession number(s) can be found in the article/**Supplementary Material**.

## Author contributions

PC: Conceptualization, Funding acquisition, Resources, Writing – original draft, Writing – review & editing. PB: Formal Analysis, Writing – review & editing. MM: Formal analysis, Investigation, Methodology, Software, Writing – review & editing. TI: Investigation, Methodology, Software, Writing – review & editing. YA: Conceptualization, Supervision, Writing – review & editing. SH: Supervision, Writing – review & editing. AK: Conceptualization, Writing – review & editing.

## Footnotes

1
https://www.bioinformatics.babraham.ac.uk/projects/fastqc/

2
https://github.com/rrwick/Porechop

3
https://github.com/wdecoster/NanoPlot

4
http://clermontyping.iame-research.center/index.php
